# Critical Success Factors (CSFs) for the Adaptive Reuse of Industrial Buildings in Hong Kong

**DOI:** 10.3390/ijerph15071546

**Published:** 2018-07-21

**Authors:** Yongtao Tan, Chenyang Shuai, Tian Wang

**Affiliations:** Department of Building & Real Estate, The Hong Kong Polytechnic University, Hong Kong 999077, China; chenyang.shuai@polyu.edu.hk (C.S.); evelyn.wang@connect.polyu.hk (T.W.)

**Keywords:** adaptive reuse, industrial buildings, critical success factor, principal component analysis, Hong Kong

## Abstract

With the economic restructuring during the 1980s and 1990s in Hong Kong, most manufacturing plants were relocated to China and many industrial buildings were left neglected or vacant. At the same time, owing to limited land supply, a shortage of affordable housing has been a problem in Hong Kong for many years. Adaptive reuse of industrial buildings may be a way of solving this problem. However, adaptive reuse is not an easy decision because there are many factors affecting adaptive reuse. Therefore, this paper examines the current situation of adaptive reuse of industrial buildings in Hong Kong and identifies a list of factors affecting the adaptive reuse of industrial buildings. Six factors are considered Critical Success Factors (CSFs). Based on a Principal Component Analysis, 33 factors are grouped into eight principal components, namely, sustainability, economics and finance, the market, changeability, location and neighborhood, culture and public interests, legal and regulatory matters, and the physical condition of the building. The identified CSFs and principal factors provide a useful reference for various stakeholders to have a clear understanding of the adaptive reuse of industrial buildings in Hong Kong, especially for the government to review current policies of adaptive reuse.

## 1. Introduction

**Figure 1:**
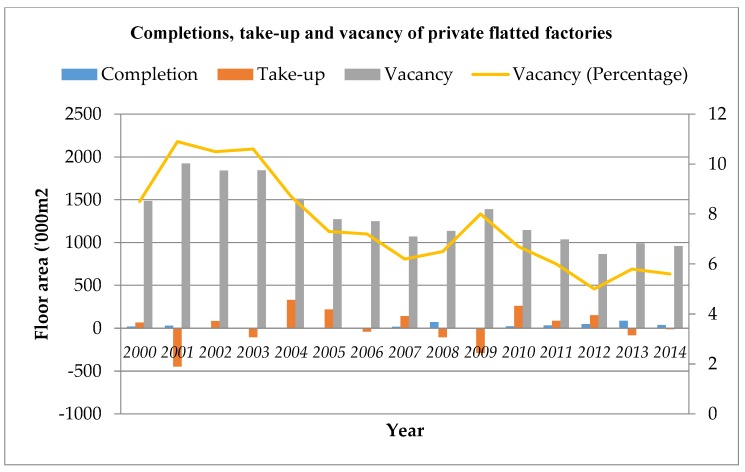
Completions, take-up, and vacancy of private flatted factories (2000–2014) *(Source: Hong Kong* *Property* *Review, 2005–2015)*.

With industrial restructuring in Hong Kong, most manufacturing plants were relocated to South China during the 1980s and 1990s. This has led to a high vacancy rate in industrial buildings, as shown in Figure 1. Almost all industrial buildings in Hong Kong are privately owned. The term ‘industrial building’ normally refers to private industrial buildings. The private industrial buildings in Hong Kong are classified into four categories, including flatted factories (refers to “an industrial building of more than one story, usually with two or more goods lifts, and constructed or converted for multiple occupation. The building is sub-divided into small, separately occupied units which are used for manufacturing, assembly and associated storage” [1], industrial/office buildings, specialized factories, and storage. The stock in the flatted factories sector was 16,850,800 m^2^ at the end of 2015, which was around 70% of the total stock of industrial buildings in Hong Kong. The vacancy in the flatted factories sector accounts for around 85% of the total vacancy of industrial buildings in Hong Kong [2]. Therefore, the vacancy in the flatted factories sector is representative.) At the end of 2001, the vacancy of private flatted factories was 1,923,000 square meters, representing 10.9% of the total stock [2]. The completion rate of industrial buildings has been very low in recent years. The vacancy rate decreased due to the implementation of revitalization measures.

In Hong Kong, the revitalization of industrial buildings was proposed in the 2009–2010 Policy Address [3]. The Planning Department carried out a survey of existing industrial buildings from April 2013 to July 2014 and issued the “Report on 2014 Area Assessments of Industrial Land in the Territory.” The report stated that there is a total of 1448 existing private industrial buildings (IBs) in all 75 industrial areas, including 476 IBs within the “I” (Industrial), 788 IBs within “OU(B)” (Other Specified Uses (Business)), 21 IBs within “R(A)” (Residential (Group A)), 116 IBs within “R(E)” (Residential (Group E)), and 47 IBs within “CDA” (Comprehensive Development Area). From January 2001 to April 2015, 295.4 ha of industrial land was rezoned to “OU(B)” or other uses [4]. All these measures encourage the adaptive reuse of existing IBs to better match the market demand. Therefore, there is a need to examine the critical factors affecting the adaptive reuse of existing IBs in Hong Kong.

This need is becoming increasingly important, since buildings consume up to 30% of primary energy and are responsible for over 30% of anthropogenic greenhouse gas (GHG) emissions [5]. Greenhouse gas emissions (GGE) in Hong Kong were 43.1 million tons (CO_2_ equivalent) in 2012 (http://www.epd.gov.hk/) and Hong Kong is considered to be the largest producer of GGE per square meter in the world. At the same time, a housing shortage problem exists in Hong Kong due to the limited land for development [6]. Adaptive reuse of industrial buildings has a shorter delivery time compared with new buildings and this option also makes corresponding contributions to sustainable development, such as GGE reduction and waste reduction [7,8,9]. An awareness of the need for building adaptation is increasing around the world and relevant policies are becoming the main drivers, such as the ‘1200 Buildings Program’ in Melbourne [10], and the ‘Wholesale Conversion of Industrial Buildings’ scheme in Hong Kong [3]. Adaptive reuse projects can be found in the United States, Australia, and across the Asia Pacific region [11,12,13]. Therefore, instead of demolition and redevelopment, adaptive reuse of buildings should be considered by key stakeholders in order to meet sustainable development goals. The paper aims to: (1) examine the current situation of adaptive reuse of industrial buildings in Hong Kong; (2) identify CSFs affecting adaptive reuse of IBs in Hong Kong; and (3) group the factors into principal components by using Principal Component Analysis (PCA).

## 2. Literature Review

As with any established metropolis, with economic and social development, existing buildings can become obsolete or disused. These buildings can be demolished for redevelopment or considered for reuse, which was described as ‘urban ore’ [14]. There are various definitions of adaptive reuse. From these definitions, it can be seen that ‘adaptive reuse’ is a special form of refurbishment that converts existing buildings into other uses.

*“Even more effective, rather than extracting these raw materials during demolition or deconstruction and assigning them to new applications, is to leave the basic structure and fabric of the building intact and change its use. This approach is called adaptive reuse”*.

[15]

*“A process that changes a disused of ineffective item into a new item that can be used for a different purpose”*.

[11]

*“Conversion of a building to undertake a modified change of use required by new or existing owners”*.

[16]

A building’s operational and commercial performance will decrease over time until it is below the expectations of owners and occupiers alike [17]. In responding to declining performances, owners and occupiers have to make decisions to either demolish and rebuild or convert existing buildings into other uses. The residual utility and value of buildings could be optimized by adaptive reuse, and demolition can be avoided [18]. The residual lifecycle expectancy can be fully exploited by adaptive reuse and it achieves a sustainable use of buildings [19]. Adaptive reuse extends the building’s life and avoids demolition waste, encourages reuse of embodied energy, and embraces the different dimensions of sustainability, including economic, social, and environmental [20,21,22,23,24,25,26,27,28,29]. Adaptive reuse-related policies have already been implemented in some countries, such as the United States and Australia [15]. Governments are seeking possible ways to reduce the overall costs of continued urban development and expansion [11].

Adaptive reuse of buildings has been successfully applied in many cities and is seen as fundamental to sustainable development, e.g., in Atlanta, GA, USA [30], Canada [31], Australia [32], and Hong Kong [33]. Particularly in metropolitan cities in the USA, such as Los Angeles, New York ,and San Francisco, adaptive reuse of commercial buildings had become a popular strategy for regeneration [19]. In Australia, adaptive reuse of buildings has a major role to play in the sustainable development of Australian communities [11]. The City of Melbourne launched the “1200 Buildings Programme” in 2010, which will lead to 1200 building adaptations before 2020 (approximately 150 per annum) [34]. Eventually, the improvement of quality of life, revitalization of neighborhoods, enhancement of economic growth, reduction of resource consumption, and negative environmental impacts as well as the preservation of historical and cultural value are supposed to be achieved through the regeneration and adaptive reuse [19].

For adaptive reuse, governments play an active and important role in regulation and expanding stakeholder knowledge of sustainable development [35]. Adaptive reuse of existing buildings involves uncertainties and risks, especially with older buildings, but in some cases, higher returns can be made by innovative building renovation [26]. Adaptive reuse extends the lifespan of the building and reduces demolition waste and its carbon footprint. Therefore, green design principles should be integrated into the adaptive reuse design for maximum reuse of the existing building components [36]. Furthermore, Langston et al. [15] developed an adaptive reuse potential (ARP) model to rank existing buildings according to their adaptive reuse potential. Tan et al. [37] developed a fuzzy adaptive reuse selection model to help decision-makers assess different alternatives for the adaptive reuse of industrial buildings.

However, the limitations of adaptive reuse were also discussed by some scholars. For example, O’Donnell [38] states that few adapted buildings could match sustainability standards so that building owners could not see the economic benefits of adaptive reuse. Certainly, the performance of an adapted building can hardly compete with that of a new building; for example, there is a short life-cycle expectancy to the existing materials and high maintenance costs. With regard to the environmental performance, it is easier for new buildings to meet green standards, starting from the design stage [27]. Some building owners and practitioners are not even willing to consider adaptive reuse because there are many risks and uncertainties [27,39]. This is despite the fact that the advantages of adaptive reuse have been widely recognized and accepted. However, building owners, users, and practitioners, in reality, only support this sustainability strategy in a piecemeal manner within certain cities [40]. Therefore, there is a need to examine the factors affecting adaptive reuse of buildings and see what might be the key to changing resistant views.

## 3. Adaptive Reuse of Industrial Buildings in Hong Kong

**Figure 2:**
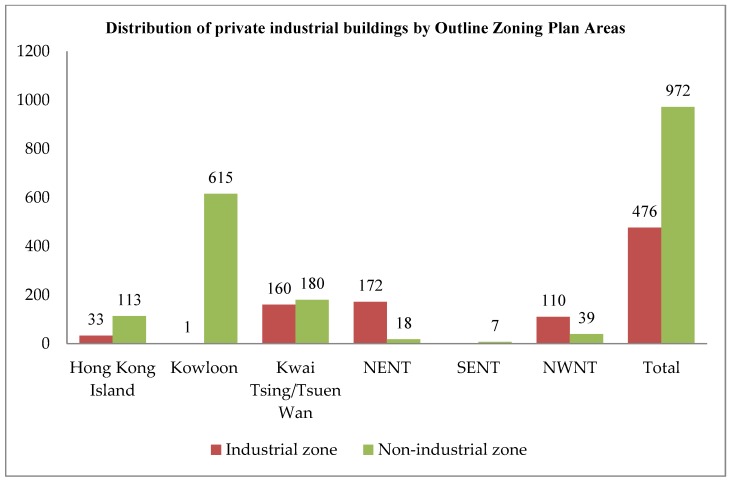
Distribution of private industrial buildings by Outline Zoning Plan Areas (Source: Planning Department, 2015 Report on 2014 Area Assessments of Industrial Land in the Territory).

**Figure 3:**
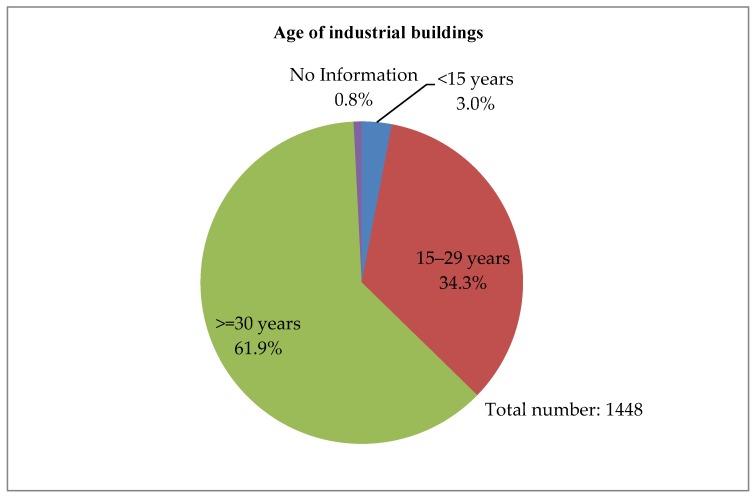
Age of industrial buildings (Source: Planning Department, 2015 Report on 2014 Area Assessments of Industrial Land in the Territory).

**Figure 4:**
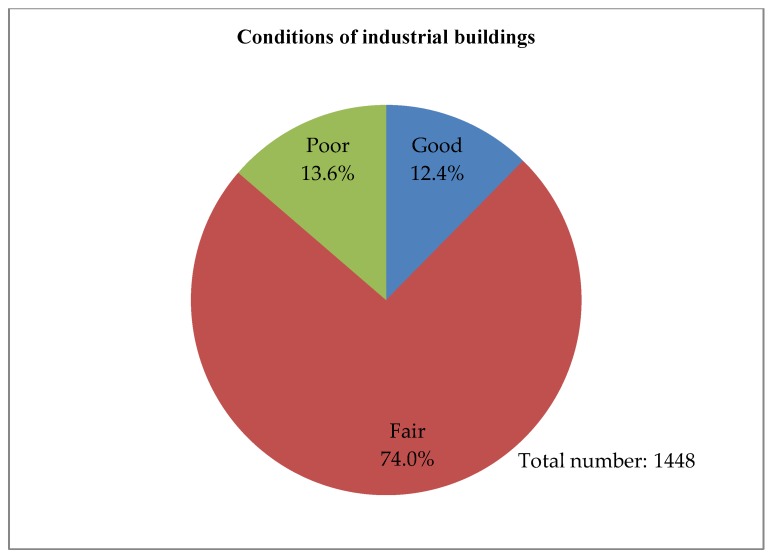
Conditions of industrial buildings (Source: Planning Department, 2015 Report on 2014 Area Assessments of Industrial Land in the Territory).

**Figure 5:**
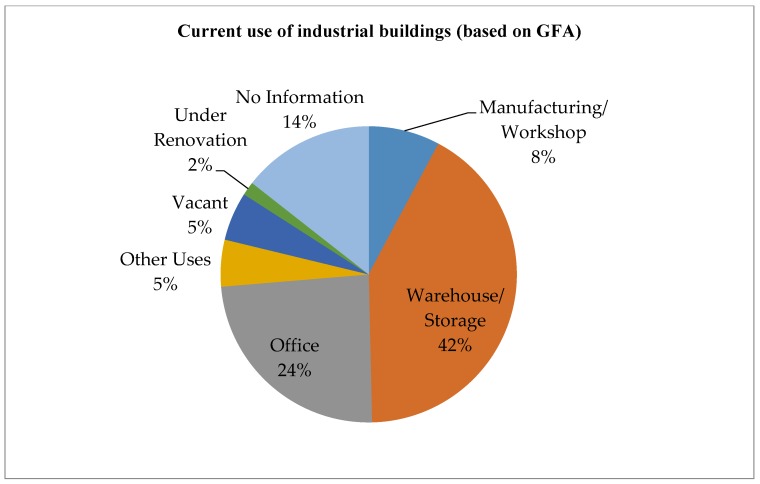
Current use of industrial buildings (based on GFA) (Source: Planning Department, 2015 Report on 2014 Area Assessments of Industrial Land in the Territory).

With the relocation of traditional manufacturing plants to Mainland China, most industrial buildings in Hong Kong has been converted for other uses [41]. At the end of 2014, the vacancy of private flatted factories in Hong Kong was 959,000 square meters, with a vacancy rate of 5.6%. Furthermore, there are a total of 1448 industrial buildings in Hong Kong, and around 67% are now situated in non-industrial zones, as shown in Figure 2 [4]. Most of the industrial buildings are in good or fair condition, and are mainly used as warehouses, storage, and offices, as shown in Figure 3, Figure 4 and Figure 5. These buildings also have large floor plans high ceilings, a strong floor loading capacity, wide corridors, and large lifts [41].

However, the conversion of existing industrial buildings for other uses was slow between 2001 and 2009, and only three cases involved wholesale conversion. Consequently, the government issued a set of revitalization measures to facilitate the redevelopment and wholesale conversion of old industrial buildings starting in 2010 [42]. These measures have activated the conversion of industrial buildings, as shown in Table 1. High property prices result in non-compliant uses of industrial buildings in Hong Kong [42]. Adaptive reuse of existing industrial buildings provides a quick solution to the problem. Furthermore, the concept of sustainability can be extended to adaptive reuse of industrial buildings with innovative solutions in line with current building regulations [43]. For example, Chai Wan Factory Estate, constructed in 1959, was successfully converted into public rental housing (Wah Ha Estate), which is a good example of enhancing public awareness of heritage conservation and sustainable housing development [44].

However, there are still several challenges to the adaptive reuse of industrial buildings in Hong Kong [45]:

(a) Multiple ownership in flatted industrial buildings makes it difficult for all owners to reach a decision to convert or redevelop the buildings;

(b) Owners are deterred by the requirement to pay full market premium for lease modification for redevelopment to other uses, or full waiver fees in the event of conversion to other uses;

(c) Building owners may be reluctant to be the “first mover” to convert or redevelop their buildings for non-industrial uses before similar uses emerge in the neighborhood; and

(d) Owners may find it difficult to raise funds for redevelopment or conversion.

There is great potential for the adaptive reuse of existing industrial buildings in Hong Kong. However, there are many factors affecting adaptive reuse decisions [40,46,47,48]. For example, Wang and Zeng [49] (2010) used six criteria for reuse selection of historic buildings, including cultural, economic, architectural, environmental, social aspects, and continuity. Wilson (2010) [50] developed selection criteria for evaluating adaptive reuse of industrial buildings in Toronto, with five adaptive reuse characteristics, including environmental, location, legislative, financial, and market characteristics. Bullen and Love [51] (2011) identified the major drivers of and barriers to the adaptive reuse of buildings. Yap [52](2013) examined the adaptive reuse potential of industrial buildings in Hong Kong and identified six factors, including market needs, developer’s risk, micro-environment suitability, financial incentives, government guidelines, and regulatory relaxation. Kee [6] examined the opportunities and constraints in converting industrial buildings to residential units with four considerations, including planning regulation and government incentives, housing affordability, design and built environment considerations, and outline zoning plan considerations.

Based on this review, a list of factors for industrial building adaptive reuse is identified in Table 2.

## 4. Research Methodology

“Success factor,” as a concept, was initially developed by Ronald [56] and the process was refined into critical success factors by Rockart [57]. There are various definitions of Critical Success Factors (CSFs). Rockart [57] proposed that CSFs are “*for any business, the limited number of areas in which results, if they are satisfactory, will ensure successful competitive performance for the organization [and] are the few key areas where ‘things must go right’ for the business to flourish.*”

Boynlon and Zmud [58] defined the CSFs as “those few things that must go well to ensure success for a manager or an organization, and, therefore, they represent those managerial or enterprise areas, that must be given special and continual attention to bring about high performance. CSFs include issues vital to an organization’s current operating activities and to its future success.”

One of the strengths of CSFs is that they allow one to reduce a large number of factors into several manageable but ‘critical’ ones so that limited resources can be allocated and aligned effectively and efficiently to maximizing profits and optimizing overall outcomes. The CSFs approach has been widely used in many research domains, including construction management [59,60,61,62,63]. Therefore, the CSFs method is used in this study to help various stakeholders have a better understanding of industrial building adaptation in Hong Kong.

Principal Component Analysis (PCA) is one of the factor extraction methods and has been used in many applications due to its simplicity and efficiency in factor extraction. There are different rotation methods in the SPSS FACTOR program [64], including Equamax, Oblimin, Quartimax, and Varimax. The Equamax rotation method was selected for the analysis in this study because this method is a compromise between Varimax and Quartimax [65], and the results are more interpretable than the other methods [66].

## 5. Data Survey

Questionnaire surveys are chosen as the primary data collection mechanism as they are effective for learning about matters that cannot be directly observed [67]. Surveys were conducted with a variety of related professionals, such as surveyors, architects, planners, engineers, project managers, and academics, who were able to contribute to this study through their tacit and explicit knowledge of the adaptive reuse of industrial buildings.

**Figure 6:**
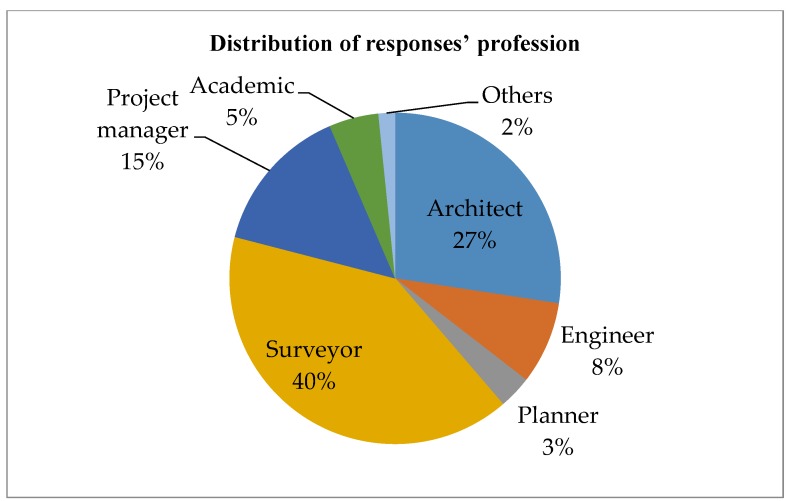
Distribution of responses’ profession.

**Figure 7:**
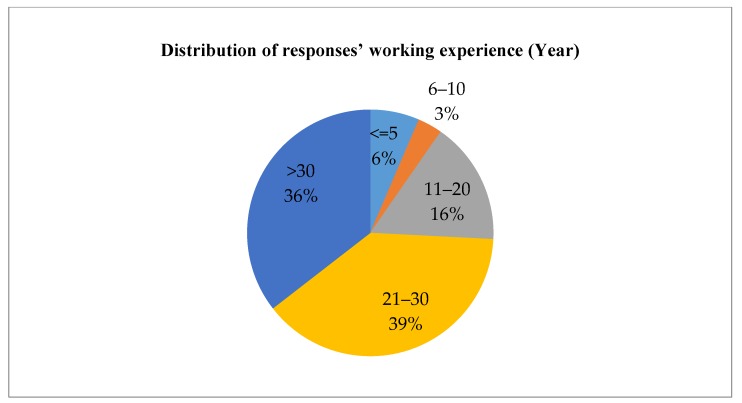
Distribution of responses’ working experience (Year).

**Figure 8:**
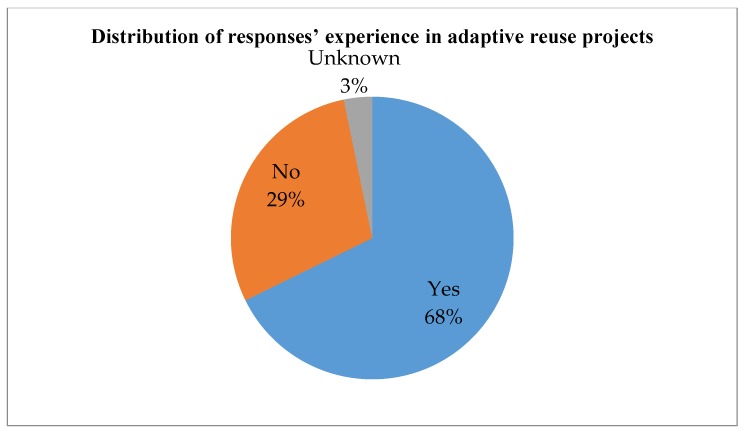
Distribution of responses’ experience in adaptive reuse projects.

A questionnaire survey was carried out in September 2015. A five-point Likert scale was used for the survey, where “5” denotes extremely important, “4” important, “3” average, “2” less important, and “1” negligible. Respondents were invited to indicate the importance of each factor identified in Table 2. Respondents were also encouraged to add factors that were not included in the original questionnaire. The respondents were registered professionals in the Hong Kong construction industry, including architects, engineers, surveyors, project managers, planners, etc. Overall, 310 questionnaires were distributed and 62 replies were received. The response rate of the survey is 20 percent, which is considered acceptable compared with the normal survey response rate in the local industry [62]. Based on preliminary analysis and data screening, 61 replies were considered valid and used in the analysis. The distribution of respondents’ professions, work experience, and experience in adaptive reuse projects is shown in Figure 6, Figure 7 and Figure 8.

The internal consistency of the collected data was tested using Cronbach’s alpha. The internal consistency is measured based on the average inter-item correlation. The Cronbach’s alpha coefficient of reliability ranges from 0 to 1. The internal consistency is considered acceptable if the Cronbach’s alpha value is greater than 0.7 [68]. The minimum Cronbach’s alpha coefficient of the collected data is 0.891, indicating good internal consistency for these factors.

## 6. Data Analysis and Discussion

### 6.1. Ranking Analysis

The ranking of means is shown in Table 3. Six factors are identified as CSFs with a mean value above 4. These CSFs are discussed as follows.

*Rank No. 1.* According to Table 3, *Market demand* (F11) is ranked as the most important CSF for industrial building adaptation, with a mean value of 4.43. This indicates that market demand is the major driver of the adaptive reuse of industrial buildings in Hong Kong. Adaptive reuse provides a fast way to meet increasing demand [41]. There are some examples reflecting the increasing demand, such as the Outline Zoning Plans (OZPs) of S/K13/26 of the Kowloon Bay and Ngau Tau Kok areas [52]. Another driver is the *commitment to sustainability***,** with the need to reduce existing buildings’ negative impact on the environment [69,70]. In the 2009–10 Policy Address, the Chief Executive outlined the direction and plans for the development of six priority industries where Hong Kong has clear advantages. These six industries include medical services, education services, environmental industries, innovation and technology, testing and certification, and cultural and creative industries [3] (HKSAR, 2009). There will be an increasing demand for office spaces from these knowledge-based industries. Adaptive reuse of existing industrial buildings would be one solution to the rising demand.

*Rank No. 3*. **Land lease control** (F30) is the third most important CSFs for the adaptive reuse of industrial buildings, with a mean value of 4.18. In Hong Kong, all land is owned by the government and leased to users for different uses and development at a premium with lease conditions. Owners need to pay a full market premium for lease modification. This is one of the challenges for private owners concerning the adaptive reuse of industrial buildings [6]. For land lease control, the Land Department has issued relevant Practice Notes to streamline the adaptive reuse applications of industrial buildings. In a Legislative Council paper “Revitalisation of Industrial Buildings” dated July 2013, the Development Bureau stated, “From the land lease perspective, wholesale conversion of an existing industrial building for “transitional accommodation” use could be effected through application for special waiver”, but “In the light of the problems outlined above, and taking into full account need to protect the well being of residents, we consider the option of ‘transitional accommodation’ not practicable ” [42]. Therefore, the government should consider relaxing the regulations on the adaptive reuse of industrial buildings in terms of land lease control.

*Rank No. 4. **Location, transportation, and accessibility*** (F02) is ranked as the fourth CSF. Location is considered one of the most important factors for developers [71]. Adaptive reuse projects are similar to new developments in terms of location. The considerations related to the location of adaptive reuse projects include the quality of the environment, safety and security, surrounding land uses, views, accessibility to services and transportation, and convenience of vehicle parking [72]. In Hong Kong, many industrial buildings are located in urban areas with good access and connectivity; some have good harbor-front views [42]. Some industrial buildings have location advantages, such as public transportation, retail, and community facilities. Therefore, the adaptive reuse potential of industrial buildings in Hong Kong is quite high.

*Rank No. 5. **Conversion cost and lifecycle cost*** (F15) is ranked as the fifth CSF. Cost is always an important concern for owners or developers for either new development or adaptive reuse. Compared with new development, adaptive reuse extends the useful life of existing buildings with lower costs in relation to materials, transport, energy, and pollution [25]. For adaptive reuse projects, there are still some uncertainties throughout the construction period, and appropriate assessments should be made to reduce additional costs in conversion [50]. However, adaptive reuse will not completely match a new building in terms of performance, and the life expectancy of an existing building is normally lower than that of a new building. The adapted building will have higher ongoing maintenance costs than a new building [73]. Therefore, lifecycle cost analysis is necessary for making effective decisions between adaptive reuse and demolition. Bullen and Love [40] pointed out that “When examining an array of building options, the building condition, scope of refit, overall cost saving, value of the building and land should be all considered for the purposes of a cost-benefit analysis.”

*Rank No. 6. **Official plan & zoning*** (F29) is ranked as the sixth CSF, with a mean value of 4.02. Adaptive reuse of existing industrial buildings may require zoning consent [37]. Changing from “Industrial” use to “Other” use is considered one of the difficulties in the adaptive reuse of industrial buildings in Hong Kong. The change of land use zoning may take a long time, which would increase the project risk [52]. There are two statutory planning controls in Hong Kong, including the Outline Zoning Plan under the Planning Department and the Building Ordinance under the Buildings Department. To encourage adaptive reuse of industrial buildings, the Town Planning Board has rezoned the suitable industrial areas from “Industrial” zones to “Other Specified Uses (Business)” zones since 2001. Most existing industrial buildings are now situated in non-industrial zones and the distribution of industrial buildings [4]. There is a need to streamline the application process and provide flexible re-zoning mechanisms for the adaptive reuse of industrial buildings.

### 6.2. Principal Component Analysis

For the extraction of individual factors, the Kaiser–Meyer–Olkin (KMO) measure of sampling adequacy and Bartlett’s Test of Sphericity are normally used [74]. The test results are shown in Table 4. The value of the test statistic for Bartlett’s sphericity is large (chi-square value 1139), the p-value is 0.000, and the value of the KMO is 0.638, which is greater than 0.5 and thus acceptable [74]. The result indicates that the correlation matrix has a mediocre degree of common variance [64]. Therefore, the sampling in this study is adequate for factor analysis.

With the collected data, factor extraction by the principal component method was conducted using the SPSS computer package. As a result, the 33 factors were grouped into eight principal components (explaining 73.96% of the cumulative total variance), as shown below:

(1) Principal Component 1 **Sustainability**
  F23 Site layout and accessibility
  F24 Material consumption
  F25 Construction waste, noise, pollution
  F26 Energy efficiency
  F27 Environmental impacts
  F28 Green design
(2) Principal Component 2 **Economics & governance**
  F13 Commercial risk and uncertainty
  F14 Financial incentives
  F15 Conversion cost and lifecycle cost
  F32 Ownership of building
  F33 Government incentives
(3) Principal Component 3 **Market**
  F11 Market Demand
  F12 Source of finance
(4) Principal Component 4 **Ease of adaptation**
  F6 Space layout
  F8 Condition of services & systems
  F9 Functional changeability
  F10 Technological difficulties
(5) Principal Component 5 **Location & neighborhood**
  F2 Location, transportation and accessibility
  F18 Compatibility with existing surroundings
  F21 Status of neighborhood
  F22 Adequacy of public facilities
(6) Principal Component 6 **Culture & public interests**
  F19 Preservation of history and culture
  F20 Health and safety concern
  F17 Public interest and support
(7) Principal Component 7 **Legal & regulatory**
  F1 Vacancy rate
  F29 Official plan & zoning
  F30 Land lease control
  F31 Building ordinance/regulations (plot ratio, fire safety, acoustic and thermal insulation,
  Daylight, escalators, etc.)
(8) Principal Component 8 **Physical condition of building**
  F3 Current use of buildings
  F4 Building age
  F5 Architectural, structural and material conditions
  F7 Condition of external fabric and finishes
  F16 Project timeline

#### 6.2.1. Sustainability

The built environment accounts for 40% of world materials usage, 40% of greenhouse gas emissions, and a third of the energy consumed by the world economy [75]. The building-in-use stage contributes 80–90% of the lifecycle energy usage in buildings, of which embodied energy contributes 10–20% [76]. Compared to new building construction, the adaptive reuse of industrial buildings generates less construction waste and makes corresponding contributions to greenhouse gas reduction [37]. Adaptive reuse of buildings can contribute to sustainability and climate change through the mitigation of CO_2_ emissions [27]. The concept of sustainability can be extended to innovative adaptation of industrial buildings with creative solutions in line with current building legislation [43]. Policy initiatives could be deployed to encourage sustainable outcomes of building adaptation [23]. For example, new applicants are encouraged to obtain certification by the BEAM Plus for wholesale conversion of industrial buildings [77]. Therefore, adaptive reuse with application of green concepts will be a better solution for vacant buildings in cities.

#### 6.2.2. Economics and Governance

The economic viability of new use is one of the barriers to successful adaptive reuse. There are some risks and uncertainties of adaptive reuse, such as the discovery of latent problems and defects [51], which may be very expensive if remedial work is needed. Building owners may not see any economic benefit to updating buildings to meet sustainability standards [38]. The capital investment could be very high in order to meet the existing building regulations, such as fire safety [52]. Therefore, financial incentives could be a driver of adaptive reuse [78]. In Hong Kong, industrial building revitalization measures were implemented in 2010. One measure is “Allowing owners to apply at a nil waiver fee for change of use of existing industrial buildings for the lifetime of the building or the current lease period, whichever is earlier. These industrial buildings should be aged 15 years or above and situated in “Industrial”, “Commercial” or “Other Specified Uses (Business)” (“OU(B)”) zones” [77]. To encourage the adaptive reuse of industrial buildings, the government should review the current policies and develop attractive financial incentives.

#### 6.2.3. The Market

The market demand is related to the potential need of adaptive reuse of industrial buildings. Hong Kong is a densely populated city and has a shortage of suitable land for housing. A housing shortage, especially of affordable housing, has existed in Hong Kong for decades [79]. Due to the high property sale and rental prices and long waiting lists for public housing, many people live in private-sector sub-divided units with poor hygiene conditions and high fire risk [6]. The demand for offices from service-based and knowledge-based industries are rising [52]. The market demand is there. Adaptive reuse can provide a fast solution for the office and housing shortage problem. However, financial uncertainty makes it more difficult for developers to secure financial backing on adaptation projects [26]. Sufficient financial resources are important to the success of an adaptive reuse project, especially for large, complex buildings. New financing methods, such as Public–Private Partnerships (PPP), should be considered to reduce the financial risks of adaptive reuse projects in the future.

#### 6.2.4. Ease of Adaptation

Adaptive reuse refers to the change the use of existing buildings for other uses than they were originally designed for. Space layout, condition of services and systems, functional changeability, and technological difficulties should be fully assessed [48].

For example, not every façade of an industrial building has windows that can meet the requirements for residential purposes. The conversion works would involve substantial alterations, which would be costly. Therefore, the conversion of industrial buildings for “Transitional Accommodation” use is not usually practical at the current stage when considering the need to protect the well-being of residents [77]. However, the adaptive reuse potential of existing industrial buildings for other uses, such as commercial offices, is still high because most industrial buildings are in good or fair condition [41]. Furthermore, new innovative and green technologies can and should be used when changing existing buildings to other uses. Relevant codes of practice and standards should be developed for the adaptive reuse of buildings.

#### 6.2.5. Location and Neighborhood

The location of buildings has long been considered the most important factor for property development. There are four factors in this principal component, including location, transportation and accessibility, compatibility with the existing surroundings, the status of the neighborhood, and the adequacy of public facilities. Most industrial buildings in Hong Kong are located in urban areas with good and improving access and connectivity [77]. The public transportation systems are well developed in Hong Kong, such as Mass Transit Railway (MTR), buses, and ferries. Many industrial buildings in Hong Kong have mixed-use ownership/tenancies. Whole conversion of industrial buildings can facilitate the revitalization of surrounding neighborhoods and change the current image and economy of the district [15,52]. Public amenities, facilities and spaces, such as public parks, and public parking, schools, and hospitals should be carefully planned for adaptive reuse projects [6]. The government should encourage more whole conversion projects that can better utilize current public facilities and revitalize the neighborhood.

#### 6.2.6. Culture and Public Interest

Older buildings can contribute to the culture of a society and preservation of these buildings can maintain their intrinsic heritage and cultural values [15]. Adaptive reuse can retain existing factory blocks and promote heritage conservation [6]. Some old industrial buildings are witnesses to the history of manufacturing in Hong Kong. On the negative side, non-compliant usage of industrial buildings is widespread in Hong Kong and fire safety is a major concern [42]. For the safety of occupants, contamination assessments should also be carried out for adaptive reuse projects [6]. Public interest and support are also important for promoting adaptive reuse. Public participation is important to get a community consensus view in support of building adaptation and optimize the building utilization after conversion. For the preservation of local culture, designers should consider how to integrate new elements into the existing culture, which is also a challenge for designers.

#### 6.2.7. Legal and Regulatory Matters

In Hong Kong, there are three types of development control: planning, building, and lease control. For the adaptive reuse of industrial buildings, the change of zoning from “Industrial” to “Other” uses is the first step. In Hong Kong, the re-zoning of the industrial areas started in 2001. Up to April 2015, about 200.3 ha and about 95.1 ha of “I” land was re-zoned to “OU(B)” and other non-industrial uses respectively [4]. This encourages more applications for the wholesale conversion of industrial buildings. For building control, the new use of the building should meet the requirements of the existing Building Ordinance. For example, the provision of natural lighting and ventilation is required for domestic buildings, and most industrial buildings do not meet these requirements. A change of regulations is not easy and is subject to the approval of the Legislative Council. Therefore, there is a need to find other possible solutions to meet the requirements of the existing Building Ordinance. For lease control, the government has implemented some measures to streamline the application process and allow tailor-made lease modifications. Further relaxation of the relevant regulations and more flexibility in land use should be considered by the government [52].

#### 6.2.8. Physical Condition of Building

Factors in this group are mainly relating to the physical characteristics of a building, such as building age, current usage, architectural, structural and material conditions, condition of external fabric and finishes, and project timeline. The physical conditions of older buildings vary and the costs of reusing them will also differ. The assessment of a building’s physical condition is important and should involve a detailed survey of the building. The deteriorated structure and fabric of buildings may require high levels of maintenance and repair, which may result in high capital cost. In this case, adaptive reuse may not be a viable option [51]. In Hong Kong, most industrial buildings are relatively young and in fair structural condition, as shown in Figure 3 and Figure 4 [4]. Most industrial buildings are for mixed use. Adaptive reuse of these buildings should mostly meet the market needs and optimize the utilization of spaces. The project timeline is also important for the success of reuse because a long project timeline increases the risks for developers [52]. A detailed survey and assessment of existing buildings should be made in a timely manner, to avoid time overruns of adaptation projects. The government should consider providing relevant consulting services on the assessment of existing buildings to building owners who are interested in the adaptive reuse of buildings.

## 7. Conclusions

With the transformation of Hong Kong’s economic structure over a period of 30 years, most manufacturing factories in Hong Kong were moved to China. This resulted in many of the existing industrial buildings being obsolete or under-utilized. However, most industrial buildings in Hong Kong are relatively young and in fair condition. Also, building adaptation provides an effective and sustainable solution for re-using these industrial buildings. Thus the useful building lifespan can be further extended through adaptive reuse to meet the increasing market demand from other sectors, such as service-based industries. In support of this transition, the Hong Kong government has implemented a set of revitalization measures to encourage the wholesale conversion or redevelopment of existing industrial buildings. However, the adaptive reuse of industrial buildings is affected by many factors, such as town planning zone allocation, building regulations, building age, current use, and accessibility. Thus, prior to this study, there has been a need to identify and examine the critical factors affecting adaptive reuse.

Based on a questionnaire survey, six critical success factors are identified, including market demand; building ordinance/regulations; land lease control; location, transportation and accessibility; conversion cost and lifecycle cost; and official planning and zoning. Government, building owners, investors, and other parties can focus on these critical factors in future adaptive reuse practices. Furthermore, the 33 factors are grouped into eight principal components by using factor analysis. The findings provide a useful reference for various stakeholders to make better decisions on the adaptive reuse of industrial buildings in Hong Kong. In future research, case studies and comparative studies in other countries/jurisdictions can explore more innovative and sustainable solutions for the adaptive reuse of buildings.

## Figures and Tables

**Table 1:** Applications under revitalization measures (as of October 2015) (Source: Hong Kong Government website: Revitalizing Industrial Buildings).

Status	Wholesale Conversion	Redevelopment	Total
Applications received	153	22	175
Approved	97	19	116
executed	66	8	74
withdrawn by applicants after approval	12	7	19
terminated after execution	12	0	12
pending execution	7	4	11
Under processing	33	3	36
withdrawn by applicants during processing	18	0	18
Rejected due to not meeting the eligibility criteria	5	0	5

**Table 2:** List of factors affecting adaptive reuse of industrial buildings in Hong Kong.

Code	Factor	References
F1	Vacancy rate	[53] [35] [23] [54] [25] [46] [47] [33] [55] [48] [43] [40] [50] [51] [52] [6] [29] [39]
F2	Location, transportation and accessibility	
F3	Current use of buildings	
F4	Building age	
F5	Architectural, structural and material conditions	
F6	Space layout	
F7	Condition of external fabric and finishes	
F8	Condition of services & systems	
F9	Functional changeability	
F10	Technological difficulties	
F11	Market Demand	
F12	Source of finance	
F13	Commercial risk and uncertainty	
F14	Financial incentives	
F15	Conversion cost and lifecycle cost	
F16	Project timeline	
F17	Public interest and support	
F18	Compatibility with existing surroundings	
F19	Preservation of history and culture	
F20	Health and safety concern	
F21	Status of neighborhood	
F22	Adequacy of public facilities	
F23	Site layout and accessibility	
F24	Material consumption	
F25	Construction waste, noise, pollution	
F26	Energy efficiency	
F27	Environmental impacts	
F28	Green design	
F29	Official plan & zoning	
F30	Land lease control	
F31	Building ordinance/regulations (plot ratio, fire safety, acoustic and thermal insulation, daylight and lift/escalator etc.)	
F32	Ownership of building	
F33	Government incentives	

**Table 3:** Ranking factors affecting adaptive reuse of industrial buildings.

Ranking	Code	Factor	Mean	SD
1	F11	Market demand	4.43	0.8458
2	F31	Building ordinance/regulations (plot ratio, fire safety, acoustic and thermal insulation, daylight and lift/escalator etc.)	4.37	0.7584
3	F30	Land lease control	4.18	0.9576
4	F02	Location, transportation and accessibility	4.16	0.9519
5	F15	Conversion cost and lifecycle cost	4.16	0.9862
6	F29	Official planning and zoning	4.02	0.9915
7	F09	Functional changeability	3.95	0.8252
8	F13	Commercial risk and uncertainty	3.89	0.8583
9	F33	Government incentives	3.89	1.1269
10	F32	Ownership of building	3.85	1.0776
11	F12	Source of finance	3.84	1.0517
12	F14	Financial incentives	3.77	1.0862
13	F06	Space layout	3.75	0.9930
14	F05	Architectural, structural and material conditions	3.72	0.8847
15	F10	Technological difficulties	3.70	0.7820
16	F23	Site layout and accessibility	3.69	0.7862
17	F20	Health and safety concern	3.58	0.9506
18	F04	Building age	3.51	0.8088
19	F18	Compatibility with existing surroundings	3.51	1.0428
20	F22	Adequacy of public facilities	3.49	0.8291
21	F16	Project timeline	3.48	0.8681
22	F27	Environmental impacts	3.43	0.8843
23	F17	Public interest and support	3.41	0.9726
24	F01	Vacancy rate	3.33	1.0361
25	F19	Preservation of history and culture	3.25	0.9426
26	F21	Status of neighborhood	3.23	0.8245
27	F26	Energy efficiency	3.18	0.9112
28	F28	Green design	3.16	0.9162
29	F03	Current use of buildings	3.08	1.0999
30	F25	Construction waste, noise, pollution	3.07	0.9105
31	F08	Condition of services and systems	3.05	0.9099
32	F07	Condition of external fabric and finishes	3.02	0.9112
33	F24	Material consumption	2.95	0.7462

**Table 4:** KMO and Bartlett’s test.

Kaiser–Meyer–Olkin Measure of Sampling Adequacy	Bartlett’s Test of Sphericity	
0.638	Approx. Chi-Square	1139
	Df	528
	Sig.	0.000
